# Nutritional status and quality of life in diabetic patients on hemodialysis: a cross-sectional study from Palestine

**DOI:** 10.1186/s41043-021-00255-w

**Published:** 2021-07-05

**Authors:** Eba’a Hafi, Ro’ya Soradi, Sarah Diab, Ahmad M. Samara, Marah Shakhshir, Malik Alqub, Sa’ed H. Zyoud

**Affiliations:** 1 Department of Medicine, College of Medicine and Health Sciences, An-Najah National University, Nablus, 44839 Palestine; 2 Public Health Department, College of Medicine and Health Sciences, An-Najah National University, Nablus, 44839 Palestine; 3 Department of Anatomy, Biochemistry and Genetics, College of Medicine and Health Sciences, An-Najah National University, Nablus, 44839 Palestine; 4 Department of Clinical and Community Pharmacy, College of Medicine and Health Sciences, An-Najah National University, Nablus, 44839 Palestine; 5 Poison Control and Drug Information Center (PCDIC), College of Medicine and Health Sciences, An-Najah National University, Nablus, 44839 Palestine; 6 Clinical Research Center, An-Najah National University Hospital, Nablus, 44839 Palestine

**Keywords:** Nutritional status, Malnutrition, Quality of life, Diabetes mellitus, Hemodialysis, Palestine

## Abstract

**Background:**

End-stage renal disease (ESRD) is a leading cause of death and morbidity worldwide. Malnutrition is a common problem among hemodialysis (HD) patients that negatively impacts their prognosis and is linked to an increase in morbidity and mortality in these patients, as well as a decrease in their quality of life (QOL). In this study, we aimed to evaluate the QOL and to investigate factors that can influence it, including nutritional status, as well as socio-demographic factors, among Palestinian diabetic patients on HD therapy.

**Methods:**

This was a cross-sectional study that occurred at a large hemodialysis center in Palestine. Malnutrition was assessed by the malnutrition-inflammation scale (MIS), and the quality of life was evaluated by using the EuroQoL five-dimensional instrument (EQ-5D). Multivariable linear regression analysis was carried out to look at the effect of multiple variables on QOL.

**Results:**

A total of 118 diabetic patients on HD were included. Of these, 66.9% were male, and 60.2% were aged 60 years or higher. Having multiple comorbid diseases (*p*=0.004) and having been on HD for >4 years (*p*=0.003) were significantly associated with a higher MIS score, whereas living alone (*p*=0.037) and having been on HD for >4 years (*p*=0.002) was significantly associated with lower EQ-5D score. We also observed a significant association between the MIS score and the EQ-5D score(r=−0.616, *p*<0.001). Multiple linear regression analysis demonstrated that diabetic hemodialysis patients who lived within a family household were positively correlated with the QOL score (standardized coefficient, 0.178; 95% confidence interval (CI), 0.042 to 0.372; *p* = 0.015), and MIS score was significantly and negatively correlated with QOL scores (standardized coefficient, −0.587; 95% CI, −0.047 to −0.028; *p* < 0.001).

**Conclusions:**

We found that malnutrition was associated with a lower QOL score among diabetic patients on HD. We recommend general practitioners, dietitians, nephrologists, and nurses to make plans that pay more attention to this group of patients who show evidence of malnutrition. Patients on dialysis for ≥ 4 years, patients who live alone, and those suffering from multiple co-morbid diseases should receive special care due to their higher risk of being impacted by this problem.

## Background

End-stage renal disease (ESRD), which is the fifth stage of chronic kidney disease (CKD) [1], is a leading cause of death and morbidity worldwide [2]. In the absence of treatment, the unavoidable decrease in renal function can be extreme enough to threaten life. Therefore, all ESRD patients need renal replacement therapy, which includes kidney transplant and hemodialysis (HD), to minimize the complications of kidney disease [3]. The renal complication of diabetes mellitus (DM) is a major cause of ESRD [4]. The incidence of DM has gradually increased worldwide due to increased life expectancy, urbanization, and changes in diet and lifestyle [5, 6], which has also resulted in more complications [7].

Malnutrition is a common problem among HD patients [8]. It is multifactorial in origin, with decreased food intake and altered metabolism as major factors [9]. Malnutrition negatively impacts the prognosis and is linked to an increase in morbidity and mortality in these patients and a decrease in their quality of life (QOL). Therefore, effective intervention could improve HD patients’ QOL and reduce the mortality rates among them [10–12]. Newly reported DM cases in the West Bank were 5555 cases, and DM complications were ranked fifth among causes of death, accounting for 7.5% of all deaths. Over the last few years, the overall number of patients diagnosed with ESRD who needed HD has increased significantly in the West Bank, Palestine. In 2018, there were 2071 patients diagnosed with ESRD and in need of HD, which constituted a continuing rise from 687 patients in 2014 and 1216 patients in 2017 [13].

Several studies have been conducted around the world to assess the quality of life and nutritional status of patients with HD, but few of these focused on diabetic patients [14–18]. In Palestine, no such studies have been conducted on diabetic patients to date. Therefore, this study aims to evaluate the QOL and to investigate factors that can influence it, including nutritional status, as well as socio-demographic factors, among Palestinian diabetic patients on HD therapy. This study underscores the importance of malnutrition and its effect on the QOL in patients undergoing HD. Understanding the nutritional effects on QOL can help inform healthcare providers about intervention strategies and programs to enhance the nutritional status of patients with HD, thus helping these patients overcome many psychological and physical problems. As performing physical activity regularly affects the nutritional status positively and may facilitate the anabolic effects of nutritional interventions. This strategy needs to be implemented and represents a promising field of investigation in all stages of CKD [19]. Moreover, in univariate analysis, the physical activity level of dialysis patients was linked to several nutritional indicators, including serum albumin concentration, serum creatinine concentration, and phase angle, and in multivariable analysis, serum albumin concentration, and lean body mass. The correlation between activity levels and these nutritional status markers is particularly intriguing since low albumin, creatinine, and phase angle have all been linked to increased mortality in dialysis patients. Low physical activity has been linked to increased mortality in the general population. It is possible that poor nutritional status leads to higher mortality, at least partly via reductions in physical activity levels [20], and about psychological problems, albumin levels were found to have a major relationship with IL-6 and depression ratings. These findings indicate that albumin and IL-6 levels in the blood could be laboratory markers linked to the presentation of emotional symptoms in hemodialysis patients. Moreover, several studies have found a connection between psychosocial variables, such as depression and hemodialysis patients’ nutritional status, specifically serum albumin concentration [21–25]. This study also provides the basis for designing models for monitoring risk factors of malnourishment in diabetic patients on HD and assessing nutritional status whenever appropriate, which would result in better dietary intervention and prevention of nutritional deficiencies.

## Methods

### Study design

This study employed a descriptive, cross-sectional design to investigate the quality of life and nutritional status among diabetic patients receiving long-term HD.

### Study setting and population of the study

This study was conducted at the department of hemodialysis in the An-Najah National University Hospital (NNUH) in Nablus city, which served as the main hemodialysis facility for the population of northern West Bank, Palestine. We used the convenience sampling technique by inviting all patients who attended this unit between October 2019 and March 2020 and met our inclusion requirements to participate in the study.

### Sample size and sampling technique

A total of 150 HD patients were scheduled to receive HD in our department. We used the online sample size calculator by Raosoft, Inc., to calculate the sample size that we needed to include in order for our study to be representative of our target population, and the result we received was 109. We subsequently approached 124 of these HD patients and asked them to participate in our study. The sampling method that we used for the subjects’ recruitment was the convenience sampling technique. We could not reach the full number, and the reason for that is the COVID-19 pandemic crisis that prevented us from reaching the An-Najah National University Hospital.

### Inclusion and exclusion criteria

The conditions for inclusion were to be aged 18 years or above, to have been undergoing regular HD for at least 6 months, and to have been diagnosed with type 2 DM with the diagnosis being documented in the participant’s medical file for more than 1 year. We excluded participants who were unable to answer the questions due to the lack of physical or mental capacity to give consent and communicate.

### Data collection instrument

The structured questionnaire used in this study was based on previously published studies [26–31] and contained four sections. The first section included socio-demographic factors (e.g., sex, age, weight, height, type of living, level of education, marital status, occupation, and household income). In the second section, we included items on the participant’s clinical condition (time since HD was started, average duration of HD session, number of sessions per week, kidney transplantation history, other chronic diseases, number of chronic medications used, smoking history and amount of cigarettes smoked, and time since DM was diagnosis).

The third section includes the MIS tool, which consists of 10 items, each addressing a certain aspect of the malnutrition-inflammation complex. Seven items were subjective global assessment (SGA) items that addressed weight change, dietary intake, gastrointestinal symptoms, functional capacity, and the presence of comorbidities, as well as signs of loss of subcutaneous fat and muscle wasting as evident by physical examination. The remaining 3 items were on body mass index (BMI) and two biochemical parameters: serum albumin level and total iron-binding capacity (TIBC) or transferrin. Each item could be answered on a four-level scale based on severity, with 0 signifying the aspect being tested was normal and three designated as being severely abnormal. The summation of all numbers a participants answered represented the Malnutrition-Inflammation Score (MIS), which ranges from 0 to 30, with a higher score signifying a more severe degree of malnutrition and inflammation. The scores were calculated using an online calculator available at the following link: http://www.touchcalc.com/calculators/mis. It is well known that the mnemonic **ABCD** summarizes the four primary components of nutritional assessment: **A** for anthropometric measurements like height and weight; **B** for biochemical parameters like serum albumin level and hemoglobin count; **C** for clinical evaluation, which includes functional, social, and mental state assessment, medical history, and physical examination; and **D** stands for dietary history, including supplement use and diet adequacy [32].

The fourth and final section contained the validated Arabic version of the European Quality of Life Scale 5 dimensions (EQ-5D) scale, which was used to measure the QOL of the participants. The EQ-5D addressed 5 dimensions of health: self-care, mobility, daily activities, anxiety and/or depression, and pain and/or discomfort. Participants were asked to answer by selecting one of the five available levels that represented how he or she felt. The five levels signified the following levels of impact: no problem at all, slightly problematic, moderately problematic, severely problematic, and extremely problematic. The European Quality of Life visual analogue scale (EQ-VAS) was also included in this section to calculate the subjects’ viewpoints on their QOL using a 100-point scale [27]. The Arabic version [28, 31, 33–37] of EQ-5D was used in accordance with the developers’ guidelines for this tool and with their permission (ID: 35675). EQ-5D score was generated according to the United States General Population Score Algorithm (i.e., EQ-5D-5L Crosswalk Index Value Calculator) to measure the index value from the value set (weights) [38]. Internal consistency for EQ-5D scale was good, with a Cronbach’s alpha value of 0.833. It should be noted that the tools that were used in the search are available in both Arabic and English languages.

### Statistical analysis

The Social Sciences Statistical Package (IBM SPSS version 21) was used for data entry and analysis. Results were described as frequencies and percentages for categorical variables and as means and SD or medians and lower-upper quartiles for continuous variables. Kolmogorov-Smirnov test was used to check the normality of data. Therefore, the Mann-Whitney test and the Kruskal-Wallis test were used as appropriate. The level of significance was set to a p value <0.05. We also tested the correlations of MIS with both EQ-5D and EQ-VAS scales, as well as the correlation between the two quality of life scales (EQ-5D and EQ-VAS), and reported r values and p values for each correlation. A multiple linear regression model, in which MIS score was considered the independent variable, was implemented to identify the factors associated with quality of life. In the multivariable linear regression analysis, variables with a p value below 0.05 in the univariate analysis and possible confounding factors were included to identify factors influencing QOL in patients with diabetic hemodialysis. Variance inflation factor (VIF) values were used to determine the multicollinearity. This model was structured to be in line with previous research [39–41]. The internal consistency reliability of the EQ-D5-5L tool was estimated by Cronbach’s alpha.

## Results

### Demographic and clinical characteristics of the participants

Out of the 124 subjects approached, 118 were included in this study, accounting for a response rate of 95.16%. The mean age of the study patients was 61.3 ± 11.2 years, and 67% of whom were males. The majority (60.2%) were ≥60 years old. Half of the subjects (50.8%) were city dwellers, and about one third (33.9%) were village dwellers, while the rest (15.3%) were living in camps. Only a minority (7%) did not receive formal education, whereas 52% completed primary education as their highest level of formal education. Most subjects (93.2%) were unemployed, with 62.7% living in a household with <2000 New Israeli Shekel (NIS) monthly income. Most of the participating patients (94.9%) were with family members or shared facilities.

The majority of subjects (55.9%) have been on HD for <4 years. Most participants underwent three sessions of HD per week (80.5%) and had sessions that persisted for <4 h (77.1%). Only 5.9% of subjects had a history of a kidney transplant. The BMI category that contained the lowest percentage of subjects (22%) was the normal weight range, with the overweight and obese BMI categories containing 39% of the participants. In addition to diabetes mellitus and ESRD, most patients were suffering from at least one additional chronic comorbid condition (e.g., hypertension), and 76.3% took five or more medications on a long-term basis. The majority of subjects (52.5%) were found to be moderately malnourished, whereas 44.1% were slightly malnourished and the remaining 3.4% suffered from extreme malnutrition. Table 1 details the socio-demographic data of the subjects, as well as their clinical characteristics.

**Table 1:** Demographic and clinical characteristics of the participants

Variable	Frequency (%); ***N*** = 118
**Age category (year)**	
<60	47 (39.8)
≥60	71 (60.2)
**Gender**	
Male	79 (66.9)
Female	39 (33.1)
**BMI category**	
Healthy weight range	26 (22.0)
Overweight range	46 (39.0)
Obese range	46 (39.0)
**Highest level of education**	
No formal education	8 (6.8)
Primary or middle school	61 (51.7)
Secondary school	32 (27.1)
Higher education	17 (14.4)
**Household monthly income**	
Low (<2000 NIS)	74 (62.7)
Moderate (2000–4999 NIS)	38 (32.2)
High (≥5000 NIS)	6 (5.1)
**Residency**	
Camp	18 (15.3)
Village	40 (33.9)
City	60 (50.8)
**Living arrangement**	
Alone	6 (5.1)
With family or others	112 (94.9)
**Marital status**	
Single, divorced, or widowed	13 (11.0)
Married	105 (89.0)
**Employment**	
Employed	8 (6.8)
Unemployed	110 (93.2)
**Dialysis vintage (years)**	
<4	66 (55.9)
≥4	52 (44.1)
**Dialysis per week**	
≤2	11 (9.3)
3	95 (80.5)
≥4	12 (10.2)
**Dialysis session duration (hours)**	
<4	91 (77.1)
≥4	27 (22.9)
**History of kidney transplantation**	
Yes	7 (5.9)
No	111 (94.1)
**Years since the diagnosis of diabetes**	
<10	16 (13.6)
10–19	32 (27.1)
≥20	70 (59.3)
**Total chronic co-morbid diseases other than diabetes**	
≤1	47 (39.8)
2	37 (31.4)
≥3	34 (28.8)
**Smoking**	
Yes	28 (23.7)
No	90 (76.3)
**Total chronic medications**	
<5	28 (23.7)
≥5	90 (76.3)
**MIS category**	
No to mild malnutrition	52 (44.1)
Moderate malnutrition	62 (52.5)
Severe malnutrition	4 (3.4)

Abbreviations: *BMI* body mass index, *NIS* New Israeli Shekel, *MIS* Malnutrition Inflammation Score

### Relationship between the participants’ characteristics and their malnutrition, inflammation, and EQ-5D scores

Table 2 shows the relationship between the participants’ demographic and clinical characteristics on the one hand and their MIS and EQ-5D scores on the other. Participants who had been on HD for ≥4 years scored significantly higher on MIS (*p* value=0.003). We also found that MIS was significantly associated with the total number of comorbid diseases other than diabetes among the participants (*p*=0.004). Regarding EQ-5D scale, participants who lived alone scored significantly higher than those who lived with their families (*p* value=0.037). Participants who had been on dialysis for ≥4 years also scored significantly higher on the EQ-5D scale (*p* value=0.002). EQ-5D was also significantly associated with the MIS category among the participants (*p* value=<0.001). No other association with either MIS or EQ-5D scale was statistically significant.

**Table 2:** Relationship between the participants’ characteristics and their Malnutrition Inflammation, and EQ-5D scores

Variable	MIS Median [Q1-Q3]	***P*** value*	EQ-5D score Median [Q1-Q3]	***P*** value*
**Age category (year)**				
<60	9.00 [7.00–10.00]	0.906 ^a^	0.61 [0.39–0.74]	0.231 ^a^
≥60	9.00 [6.00–11.00]		0.49 [0.30–0.70]	
**Gender**				
Male	9.00 [6.00–11.00]	0.814 ^a^	0.62 [0.37–0.76]	0.071 ^a^
Female	9.00 [7.00–11.00]		0.48 [0.30–0.65]	
**BMI category**				
Healthy weight range	10.00 [6.75–16.25]	0.291 ^b^	0.53 [0.21–0.73]	0.637 ^b^
Overweight range	9.00 [6.00–11.00]		0.54 [0.41–0.75]	
Obese range	8.50 [7.00–10.00]		0.54 [0.32–0.71]	
**Level of education**				
No formal education	9.00 [8.00–10.50]	0.423 ^b^	0.47 [0.21–0.63]	0.325 ^b^
Primary or middle school	9.00 [5.00–11.00]		0.50 [0.34–0.67]	
Secondary school	9.50 [7.00–14.00]		0.56 [0.33–0.72]	
Higher education	8.00 [6.50–10.00]		0.67 [0.47–0.81]	
**Household monthly income**				
Low (<2000 NIS)	9.00 [6.00–11.00]	0.610 ^b^	0.50 [0.32–0.67]	0.289 ^b^
Moderate (2000-4999 NIS)	9.00 [7.00–11.00]		0.61 [0.41–0.75]	
High (≥5000 NIS)	9.50 [5.50–14.25]		0.68 [0.32–0.86]	
**Residency**				
Palestinian refugee camps	9.00 [6.00–10.25]	0.664 ^b^	0.46 [0.35–0.63]	0383 ^b^
Village	8.50 [6.25–10.75]		0.62 [0.49–0.73]	
City	9.00 [6.00–11.75]		0.51 [0.30–0.74]	
**Living arrangement**				
Alone	11.50 [4.75–12.25]	0.580 ^a^	0.36 [0.05–0.51]	**0.037** ^**a**^
With family or others	9.00 [6.00–11.00]		0.58 [0.36–0.72]	
**Marital status**				
Single, divorced, or widowed	9.00 [6.00–11.50]	0.952 ^a^	0.45 [0.21–0.62]	0.79 ^a^
Married	9.00 [6.00–11.00]		0.57 [0.36–0.73]	
**Employment**				
Employed	8.50 [8.00–10.75]	0.872	0.64 [0.51–0.83]	0.93 ^a^
Unemployed	9.00 [6.00–11.00]		0.52 [0.32–0.71]	
**Dialysis vintage (years)**				
<4	8.00 [6.00–10.00]	**0.003** ^**a**^	0.62 [0.43–0.76]	**0.002** ^**a**^
≥4	10.00 [8.00–13.00]		0.45 [0.22–0.63]	
**Dialysis per week**				
≤2	7.00 [6.00–11.00]	0.721 ^b^	0.48 [0.26–0.67]	0.782 ^b^
3	9.00 [6.00–11.00]		0.52 [0.35–0.72]	
≥4	9.00 [7.25–11.00]		0.63 [0.35–0.73]	
**Dialysis session duration (hours)**				
<4	9.00 [7.00–11.00]	0.107 ^a^	0.57 [0.32–0.72]	0.469 ^a^
≥4	7.00 [5.00–10.00]		0.51 [0.48–0.70]	
**History of kidney transplantation**				
Yes	7.00 [7.00–10.00]	0.672 ^a^	0.50 [0.15–0.63]	0.473 ^a^
No	9.00 [6.00–11.00]		0.56 [0.35–0.72]	
**Years since the diagnosis of diabetes**				
<10	7.50 [6.00–9.75]	0.112 ^b^	0.47 [0.33–0.65]	0.351 ^b^
10–19	8.50 [6.00–10.00]		0.62 [0.39–0.72]	
≥20	9.50 [7.00–12.00]		0.54 [0.31–0.74]	
Total chronic co-morbid diseases other than diabetes**				
≤1	7.00 [6.00–10.00]	**0.004** ^**b**^	0.62 [0.37–0.81]	0.176 ^b^
2	9.00 [8.00–12.00]		0.48 [0.32–0.64]	
≥3	10.00 [7.00–13.00]		0.62 [0.28–0.71]	
**Smoking**				
Yes	10.00 [7.00–11.00]	0.286 ^a^	0.55 [0.40–0.75]	0.391 ^a^
No	9.00 [6.00–11.00]		0.53 [0.32–0.71]	
**Total chronic medications**				
<5	9.00 [6.25–11.00]	0.727 ^a^	0.64 [0.33–0.76]	0.347 ^a^
≥5	9.00 [6.00–11.00]		0.51 [0.35–0.69]	
**MIS category**				
No to mild malnutrition	-	-	0.65 [0.48–0.82]	**<0.001** ^**b**^
Moderate malnutrition	-		0.48 [0.32–0.64]	
Severe malnutrition	-		0.05 [0.02–0.11]	

Abbreviations: *MIS*, Malnutrition Inflammation Score; *EQ-5D*, European Quality of Life scale 5 dimensions; *BMI*, body mass index; *NIS*, New Israeli Shekel

*Significant *p* values are in bold

**Other chronic co-morbid diseases include hypertension, heart failure, arthritis, stroke, myocardial infarction, chronic lung disease, chronic rheumatologic disorders, and thyroid disorders

^a^Mann–Whitney U test

^b^Kruskal–Wallis test

### Correlations with QOL in diabetic patients with HD

Table 3 summarizes the correlations between the MIS score and the two QOL scores in diabetic patients with HD. Correlation analyses showed a moderate negative association between the QOL and MIS scores (*r* =−0.616; *p* value < 0.001), a moderate positive association between QOL and EQ-VAS scores (*r* = 0.489; *p* value < 0.001), and finally, a moderate negative association between MIS scores and EQ-VAS scores (*r* = −0.366; *p* value < 0.001).

**Table 3:** Correlations with quality of life in diabetic patients on hemodialysis

Scales	EQ-5D *P* value	Correlation	EQ-VAS *P* value	Correlation
**MIS**	<0.001	–0.616	<0.001	–0.366
**EQ-5D**			<0.001	0.489
**EQ-VAS**	<0.001	0.489		

Abbreviations: *EQ-5D*, European Quality of Life scale 5 dimensions; *EQ-VAS*, European Quality of Life visual analogue scale; *MIS*, Malnutrition Inflammation Score

### Results of multiple linear regression analysis

The multiple linear regression analysis was performed by using the QOL score as a dependent variable after controlling for living arrangement, dialysis vintage, and MIS score demonstrated that diabetic hemodialysis patients who lived in a family household were positively correlated with the QOL score (standardized coefficient, 0.178; 95% confidence interval (CI), 0.042 to 0.372; *p* = 0.015), and MIS score was significantly and negatively correlated with QOL scores (standardized coefficient, −0.587; 95% CI, −0.047 to −0.028; *p* < 0.001). The factors significantly associated with QOL scores based on multiple linear regression findings are illustrated in Table 4. There was no evidence of multicollinearity between independent variables (VIF ranged from 1.012-1.116).

**Table 4:** Multivariable linear regression analysis of the independent variables’ association with the quality of life in diabetic patients on hemodialysis

Model	Unstandardized Coefficients		Standardized Coefficients	t	***P*** value*	95.0% Confidence Interval for B		Collinearity Statistics
	B	Std. Error	Beta			Lower Bound	Upper Bound	VIF
	0.481	0.170		2.835	0.005	0.145	0.817	
**Dialysis vintage**	–0.043	0.039	–0.085	–1.122	0.264	–0.120	0.033	1.116
**Living arrangement,**	0.207	0.083	0.178	2.480	**0.015**	0.042	0.372	1.012
**MIS score**	–0.037	0.005	–0.587	–7.837	**0.000**	–0.047	–0.028	1.104

*Bold values denote statistical significance at the p < 0.05 level

## Discussion

In the current study, we evaluated the nutritional status and QOL of diabetic patients on HD, as well as factors associated with these two indices. Although diabetic HD patients’ QOL [18] and their nutritional status [30] were previously studied in Palestine, no previous studies investigated the relationship between these two health measures among this population. Therefore, this study was the first attempt to investigate this relationship in Palestine.

Diabetic patients on HD suffer from significantly greater complications and worse QOL compared to patients who have DM but are not on HD [42, 43]. This study used a common nutritional assessment scale called MIS, which contains four sections covering the following aspects: the patient’s medical history, physical examination, BMI measurements, and relevant laboratory test results [26].

Our results showed that 52.2% of the participants suffered from moderate malnourishment, which is above what is expected, based on findings reported in the literature. For example, Jenny et al. targeted the same group of patients and evaluated their nutritional status using the Subjective Global Assessment (SGA) questionnaire, and their QOL using the Kidney Disease and Quality of Life-Short form (KDQOL-SFTM), and reported that 48% were moderately malnourished [16].

In our sample, malnutrition was significantly associated with the patient’s vintage, dialysis, and the total number of chronic comorbid diseases. This is similar to results from a 2019 study that found that patients with long vintage dialysis had a significantly higher MIS score after a multivariable linear regression model [30], contrary to the findings from a 2004 study that reported no such correlation [44]. Another 2014 study also reported the prevalence of malnutrition and uncovered a clear association between long periods of HD and deteriorating nutritional status [45]. However, there was no association between the participants’ MIS scores and their age or sex in our study. This is similar to the findings of a 2011 study that found no significant correlation between SGA scores and neither age, not sex [46].

Another key finding in our study was that comorbidities were a significant predictor of malnutrition in diabetic patients on HD. This result is consistent with that of a study published in 2010 and reported that other comorbid diseases may influence the nutritional status by raising the catabolic rate in the body and decreasing nutritional intake, resulting in depleted energy stores [47]. Similarly, another study that used dialysis malnutrition scores and reported on protein and energy intake found that comorbidities and inflammation were predictors of malnutrition in a significant decrement order [48].

The results of this study showed that the studied variables, which included sex, BMI, age, employment, living arrangement, marital status, employment, number of weekly sessions of dialysis, and transplantation history, were not significantly associated with malnutrition in diabetic patients undergoing HD. In accordance with these results, it was reported by another cross-sectional analysis study conducted in Palestine that no significant connection between BMI and nutritional status was found [30]. In another cross-sectional study that examined serum albumin levels as an index of nutritional state in diabetic patients on HD, malnutrition prevalence was not linked to sex [49].

On reviewing the relevant literature, QOL in diabetic patients was commonly evaluated using EQ-D5 [7, 37, 50–54]. It was frequently used by patients on HD as well [31, 55–57]. Many studies reported that diabetic patients undergoing dialysis suffered substantially more complications and had worse QOL relative to patients with DM but are not on dialysis [42, 43]. In this study, the mean EQ-D5 score in diabetic patients on HD was 0.5± 0.2, and the mean EQ-VAS score of the same population was 51.6 ± 21.2, which was similar to previously reported scores in the same group in Palestine [29, 58]. However, it was noticeably lower than reported scores in studies that used the same tool in other countries such as Norway [7].

It was clear from our findings that QOL is worse in patients living alone and those suffering from multiple comorbid diseases, which is consistent with the results of previous studies [59–61]. Moreover, our findings suggest that diabetic patients on HD for >4 years had better QOL, a finding that is similar to that of another study conducted in Egypt in 2018 [62]. We found no statistically significant association between QOL and sex, monthly household income, marital status, and educational level, which is in concordance with findings from a 2010 study conducted in Finland that found no correlation between the QOL of diabetic patients on HD and their sex or socioeconomic status [43]. Similarly, another study from China showed that the level of education, source of money, and marital status did not significantly influence QOL in this group [63].

Finally, we found a strong correlation between nutritional status and QOL in diabetic patients undergoing HD. No previous studies examined the association of these two indices among diabetic patients in Palestine before. Therefore, this finding should further assess this correlation to establish a cause–effect relationship between the two measures and investigate possible solutions to both problems.

### Strength and limitations

In Palestine, the prevalence of malnutrition in diabetic patients on HD has never been studied before; neither has been the correlation between nutritional status and QOL among this particular population, making this the first study in Palestine to study these questions. Like any study, this study had some limitations, including its cross-sectional design, which limited our capacity to make causality relationships between variables. Another limitation of this study was that it occurred in a single center, potentially making its findings not generalizable for all HD patients in Palestine. Additionally, there were some clinical factors that we did not incorporate in our study and that may have influenced the nutritional state of the patients. These include the existence of residual renal function or overall weekly dialysis time.

## Conclusions

In conclusion, we found that malnutrition was associated with a lower QOL score among diabetic patients on HD. It was also found that having multiple comorbid diseases and having been on HD for >4 years were significantly associated with malnutrition, whereas living alone and having been on HD for >4 years significantly impacted QOL. We recommend that healthcare officials (e.g., clinicians, dietitians, and nurses) consider these findings and make plans that pay more attention to diabetic patients receiving HD treatment and show evidence of malnutrition to improve their QOL. Patients on dialysis for ≥ 4 years, patients who live alone, and those suffering from multiple comorbid diseases should receive special care due to their higher risk of being impacted by this problem. Additionally, efficient screening for malnutrition risk factors in diabetic patients on HD and nutritional monitoring and evaluation should also be considered to promote early dietary intervention and prevent further decline in nutritional status and QOL.

## Data Availability

The datasets used and/or analyzed in this study will be made available by the corresponding author upon reasonable request.

## Abbreviations

ESRD: End-stage renal disease
CKD: Chronic kidney disease
HD: Hemodialysis
DM: Diabetes mellitus
QOL: Quality of life
NNUH: An-Najah National University Hospital
SGA: Subjective Global Assessment
BMI: Body mass index
TIBC: Total iron-binding capacity
EQ-5D: European Quality of Life scale 5 dimensions
CI: Confidence interval
EQ-VAS: European Quality of Life Visual Analogue Scale
MIS: The Malnutrition-Inflammation Score
IRB: Institutional Review Board
